# Effect of plant‐based diets on plasma biomarkers of Alzheimer's disease

**DOI:** 10.1002/alz.086002

**Published:** 2025-01-09

**Authors:** Shaun Eslick, Grace Austin, Jessica Ferguson, Manohar Garg, Ralph N Martins

**Affiliations:** ^1^ Macquarie University, Sydney, NSW Australia; ^2^ University of Newcastle, Callaghan, NSW Australia

## Abstract

**Background:**

Interest and consumption of plant‐based diets (PBD) in the 21^st^ century continued to increase, particularly in western societies, with the perception that PBDs are associated with beneficial health outcomes and a reduced environmental footprint. Evidence suggests that PBDs may be protective against neurodegenerative diseases, such as Alzheimer’s disease (AD). Health effects of PBDs such as reduction of inflammation, shift in gut microbiota composition, reduced risk of type 2 diabetes and cardiovascular disease are all believed to attribute to reduced AD risk. Therefore, we hypothesise that plasma amyloid beta (Aβ), glial fibrillary acidic protein (GFAP), neurofilament light (NFL) and phosphorylated tau (pTau) will be lower in individuals habitually following a vegan dietary pattern compared to those following a regular meat‐eating diet.

**Method:**

Men and women, 30‐75yrs without current or diagnosed CVD who followed a vegan (nil animal flesh or animal derived products) or regular meat (≥daily consumption of animal flesh) dietary pattern for a minimum of six months. Fasting blood was collected and plasma separated by centrifugation and stored at ‐80°. Biomarkers of AD including Aβ1‐40, Aβ1‐42, GFAP, and NFL and pTau181 were measured using ultra‐sensitive Single Molecule Array (SIMOA) assay platform.

**Result:**

A total of 94 plasma samples were analysed from the vegan (n=47) and the regular meat eaters (n=47) dietary pattern groups. In the vegan group, the mean plasma Aβ1‐42/Aβ1‐40 ratio (p=0.01), plasma GFAP (p=0.04) and plasma NFL (p<0.01) were significantly lower compared to the regular meat eaters’ group. However, plasma pTau181 was found to be similar between the two groups (p=0.29).

**Conclusion:**

This study demonstrated that blood‐based biomarkers are lower in individuals following a vegan dietary pattern compared to those following a regular meat‐eating diet, suggesting that PBDs may be protective against Alzheimer’s disease pathology.

